# Draft Nuclear Genome Sequence of the Halophilic and Beta-Carotene-Accumulating Green Alga *Dunaliella salina* Strain CCAP19/18

**DOI:** 10.1128/genomeA.01105-17

**Published:** 2017-10-26

**Authors:** Juergen E. W. Polle, Kerrie Barry, John Cushman, Jeremy Schmutz, Duc Tran, Leyla T. Hathwaik, Won C. Yim, Jerry Jenkins, Zaid McKie-Krisberg, Simon Prochnik, Erika Lindquist, Rhyan B. Dockter, Catherine Adam, Henrik Molina, Jakob Bunkenborg, EonSeon Jin, Mark Buchheim, Jon Magnuson

**Affiliations:** a Department of Biology, Brooklyn College of the City University of New York, Brooklyn, New York, USA; b The Graduate Center of the City University of New York, New York, New York, USA; c Department of Energy, Joint Genome Institute, Walnut Creek, California, USA; d University of Nevada, Department of Biochemistry and Molecular Biology, Reno, Nevada, USA; e HudsonAlpha Institute for Biotechnology, Huntsville, Alabama, USA; f The Proteomics Resource Center, The Rockefeller University, New York, New York, USA; g Alphalyse A/S, Odense, Denmark; h Department of Life Science, Hanyang University, Research Institute for Natural Sciences, Seoul, Republic of Korea; i Biological Science, The University of Tulsa, Tulsa, Oklahoma, USA; j Pacific Northwest National Laboratory, Richland, Washington, USA

## Abstract

The halotolerant alga *Dunaliella salina* is a model for stress tolerance and is used commercially for production of beta-carotene (=pro-vitamin A). The presented draft genome of the genuine strain CCAP19/18 will allow investigations into metabolic processes involved in regulation of stress responses, including carotenogenesis and adaptations to life in high-salinity environments.

## GENOME ANNOUNCEMENT

The halotolerant green alga *Dunaliella salina* Teodoresco is the type species of the *Dunaliella* genus (class *Chlorophyceae*) (1, 2). The biflagellate cells grow best at salinities of about 1.5 M (3). Often, new isolates of halotolerant algae of this general appearance are incorrectly identified as *D. salina*. Strain CCAP 19/18 is a genuine representative of the species *D. salina* (4), making it a reference for all ongoing and future studies on this and other *Dunaliella* species.

Due to the absence of a rigid cell wall and the presence of a glycocalyx (5), *D. salina* cells can adjust rapidly to large changes in salinity. Thus, *D. salina* is a model for studies of salinity tolerance (6–11). Further, under abiotic stress cells turn orange due to overaccumulation of beta-carotene in plastidic oil globules (12). Beta-carotene may accumulate to over 8% of the cellular dry weight, making the alga an important crop for the health food market for production of natural beta-carotene (13–17). With the recent surge in *Dunaliella* “omics” research and currently 19 BioProjects deposited in NCBI (NIH), we offer this early release draft genome sequence of strain CCAP19/18 to support and encourage ongoing and future research.

Following genomic DNA extraction from cells, libraries were generated for sequencing using either the Illumina or the PacBio platforms, overall producing 554.53 Gbp (after quality filtering). The tight-insert protocol was used for library construction for the Illumina HiSeq 2500 paired-end (PE) runs, which produced 219.9 Gbp (after quality filtering). For Illumina HiSeq 2000 sequencing preparation, the ligation-free paired end (LFPE) construction protocol was used for four libraries, the Illumina standard PE unamplified protocol was used for three libraries, and the Illumina CLIP PE protocol was used for one library, in total providing 341.6 Gbp (after quality filtering). Sequences generated on the Illumina platform provided 62× coverage and those from paired-end reads 27× (from 2-kb, 4-kb, and 6-kb libraries). Additionally, 16 PacBio single-molecule real-time (SMRT) libraries were constructed and sequenced on the PacBio RS platform, providing, after quality filtering, a total of 3.162 Gbp and resulting in about 10× coverage. The chloroplast and mitochondrial sequences (18) were removed prior to assembly.

The genome was assembled with ALLPATHS-LG (19). The resulting assembly was scaffolded using SSPACE (20) with three fosmid libraries (total of 0.50× read coverage). The final assembly was screened for contaminants against the NCBI nonredundant database. The *D. salina* v1.0 draft genome is provided through the Phytozome portal from the U.S. Department of Energy (DOE) Joint Genome Institute (JGI) at http://phytozome.jgi.doe.gov/. The main genome assembly provides approximately 343.7 Mb arranged in 5,512 scaffolds (*N*_50_ = 353.0 kbp) and a 49.1 G+C content. The contig *N*_50_ (*L*_50_) = 10,635 (7.8 kb), and 1,241 scaffolds are >50 kb in size, representing approximately 92.0% of the genome. With a conservative annotation approach using a repeat-masked assembly, the presented draft genome contains 16,697 loci providing 18,801 protein-coding transcripts. A less conservative annotation approach without masking repeats revealed 36,851 protein-coding genes. The assembly and annotation are expected to provide the foundation for future studies on halotolerance, secondary carotenoid accumulation, and algal stress biology.

### Accession number(s).

The whole-genome sequencing project was deposited in GenBank under BioProject number PRJNA32771. The project BioSample number is SAMN02746051 (sample name 1014865, SRA number SRS1543949). A trace file archive is available under NCBI project ID 32771. The draft genome sequence is accessible under GenBank accession number NSFN00000000. This paper describes the first version, NSFN01000000.
